# Development of a prediction model for ctDNA detection (Cir-Predict) in breast cancer

**DOI:** 10.1007/s10549-025-07647-0

**Published:** 2025-03-07

**Authors:** Chiaki Nakauchi, Nanae Masunaga, Naofumi Kagara, Chiya Oshiro, Masafumi Shimoda, Kenzo Shimazu

**Affiliations:** 1 Department of Breast Surgery, ISEIKAI International General Hospital, 4-14 Minamioogimachi, Kita-ku, Osaka City, Osaka Japan; 2 Department of Breast and Endocrine Surgery, Graduate School of Medicine, Osaka University, 2-2-E10 Yamadaoka, Suita, Osaka 565-0871 Japan; 3 Department of Breast Surgery, Osaka General Medical Center, 3-1-56, Bandai-Higashi, Sumiyoshi-ku, Osaka City, Osaka 558-8558 Japan; 4 Department of Breast Surgery, Kaizuka City Hospital, 3-10-20 Ichibori, Kaizuka, Osaka Japan

**Keywords:** Breast cancer, CtDNA, Liquid biopsy, DNA microarray

## Abstract

**Purpose:**

The detection of circulating tumor DNA (ctDNA) is a valuable method to predict the risk of recurrence and to detect real-time gene changes. The amount of ctDNA is affected by many factors. Moreover, the detection rate of ctDNA varies from report to report.

**Methods:**

The present study evaluated differentially expressed genes using a DNA microarray assay for gene expression in tumors with and without detected ctDNA and constructed a prediction model for the detectability of ctDNA in breast tumor tissues. The model, named Cir-Predict, consisted of 126 probe sets (111 genes) and was constructed in a training set of breast cancer patients (*n* = 35) and validated in a validation set (*n* = 13).

**Results:**

The accuracy, sensitivity, and specificity in training and validation sets were over 90%, and Cir-Predict was significantly associated with ctDNA detection independently of the other conventional clinicopathological parameters in training and validation sets (*P* < 0.001, *P* = 0.014, respectively). Cir-Predict (+) was significantly associated with worse recurrence-free survival (*P* = 0.006). Pathway analysis revealed that nine pathways including tight junction and cell cycle tended to be related to ctDNA detectability.

**Conclusion:**

Cir-Predict not only provides information useful for breast cancer treatment, but also helps the understanding of the mechanism by which ctDNA is detected.

**Supplementary Information:**

The online version contains supplementary material available at 10.1007/s10549-025-07647-0.

## Introduction

Breast cancer is a complex and heterogeneous disease [1]. Tissue biopsy and immunohistochemistry are the gold standard techniques to guide the selection of breast cancer therapy; however, these techniques do not assess the molecular heterogeneity of tumors and are unable to capture mutations that change over time. In contrast, liquid biopsy allows the detection of specific tumor biomarkers in a real-time manner, and specimens can be obtained using a minimally invasive method. Liquid biopsy involves the analysis of circulating tumor cells, circulating tumor DNA (ctDNA), circulating tumor RNA, messenger RNA, and microRNA, which are released from the primary tumor and/or metastatic deposits into blood, urine, salvia, and other biological samples [2]. Among these liquid biopsy techniques, ctDNA has been the subject of much attention. ctDNA is detected for a wide range of purposes, such as monitoring real-time genomic or epigenomic alterations, identifying genes that are targets of treatment, determining early treatment efficacy, monitoring minimal residual disease, and evaluating real-time treatment resistance [2, 3]. Previous studies showed that ctDNA may be associated with shorter disease-free survival in both early-stage and advanced metastatic breast cancer [2, 4, 5]. Whether ctDNA can be detected in a sample is useful information.

Cell-free DNA (cfDNA) consists of fragments of DNA circulating freely in the peripheral blood, most of which are released from blood cells, erythrocyte progenitors, and vascular endothelial cells [2]. In cancer patients, cfDNA contains a small amount of tumor-derived DNA fragments, which is called ctDNA. The frequency of ctDNA in cfDNA is generally very small (< 1.0%) [6]. Because of fragmentation and a short half-life (1–2 h) [7], ctDNA is difficult to detect. However, recent advances in molecular-based technologies, including high sensitivity digital PCR (dPCR) or next-generation sequencing technology, offering a tremendous sequencing capacity with groundbreaking depth and accuracy, have enabled the detection of ctDNA 3, 8, 9. Now, ctDNA analysis has emerged as a promising tool for the management of breast cancer, offering a non-invasive approach for disease monitoring and treatment guidance. Its sensitivity is not 100%, and false-negative results can occur. Several factors have been suggested to influence ctDNA detection rates, including breast cancer subtype, tumor size, tumor burden, and other biological factors, such as tumor aggressiveness, angiogenesis, and patient's metabolic status [10].

PIK3CA mutations in cancer occur predominantly in exons 9 and 20, including H1047R, E545K, and E542K, which are called ‘‘hot-spot” mutations. These three mutations account for 70%–80% of PIK3CA mutations in cancer. We previously reported that PIK3CA mutations in serum DNA detected using dPCR for PIK3CA mutations (H1047R, E545K, and E542K) are predictive of recurrence in primary breast cancer. The sensitivity of the dPCR assay for the mutant alleles in cell lines with PIK3CA mutations was 0.01%. The detection rate of PIK3CA mutation in serum DNA was 22.7% (25/110) in primary breast cancer patients with PIK3CA mutant tumors [4]. Beaver et al. evaluated PIK3CA mutations in the plasma of breast cancer patients and the sensitivity was 93.3% and specificity was 100% for detecting early-stage breast cancer [11]. The frequency with which ctDNA is detected in early breast cancer varies among reports, but ctDNA is not detected in all early breast cancer cases. This is partly because the level of ctDNA in patients is very low and its half-life is short; moreover, the amount of ctDNA is affected by multiple factors, such as tumor location, size, metastasis, vascular infiltration, tumor status, and stage.

Recently, cfDNA-based comprehensive genomic profiling (CGP) assays have been reported as a complement to tissue-based testing to ensure potentially life-extending therapies are administered to patients. The FoundationOne®Liquid CDx assay is a pan-cancer cfDNA-based CGP assay that was approved by the FDA [12]. The rates of concordance between cfDNA-based CGP assays and tumor tissue genomic assays are not 100%. This is partly because the blood and tumor samples are collected at different times and because the ctDNA is not included in the cfDNA used for the assay. Thus, when genomic alteration information cannot be obtained, a tool that predicts the detection of ctDNA can help determine whether cfDNA is tested and not ctDNA by CGP assays or if the genomic alteration is not present in ctDNA. DNA microarray assays for the evaluation of gene expression in breast cancer tissues have been used to develop multiple gene classifiers for the prediction of recurrence [3–15] and response to chemotherapy [16, 17]. To date, there is no prediction model for ctDNA detection.

In the current study, we evaluated differences in gene expression in breast cancer tumors with and without detectable ctDNA. This analysis will provide insights into whether the gene expression of the tumor itself has a role in whether ctDNA can be detected. And we evaluated the correlation between the clinical outcomes and our predict model constructed for the prediction of ctDNA detectability. The ability of our model to accurately predict patient outcomes will provide further evidence suggesting that the presence of ctDNA is an unfavorable prognostic indicator in breast cancer.

## Materials and methods

### Patients

Forty-eight primary breast cancer patients (stage I–III) who had received no neoadjuvant systemic therapy and had undergone mastectomy or breast-conserving surgery followed by radiation therapy at Osaka University Hospital between June 2000 and November 2009 were retrospectively included in this study. Informed written consent was obtained from all patients before surgery. The study protocols were approved by the Ethics Committee of Osaka University Graduate School of Medicine (Approval Number: 11337, Date of Approval: May 7, 2012).

Digital PCR was performed in serum samples from 313 preoperative breast cancer patients with PIK3CA mutant tumors. Cases in which the same PIK3CA mutation in the tumor was found in the serum were classified as ctDNA positive; cases in which the serum did not show the same PIK3CA mutation were classified as ctDNA negative. Cases with both ctDNA analysis data and tumor microarray expression data were used to establish the training and validation sets.

The training set, which was used for ranking probes related to ctDNA detection, consisted of 25 patients with invasive breast cancer whose expression was analyzed using microarray at Osaka University Hospital in 2011 (OUH_1) and 10 patients whose expression was analyzed using microarray at Osaka University Hospital in 2012 (OUH_2). The validation set consisted of 13 patients whose expression was analyzed using microarray at Osaka University Hospital in 2015 (OUH_3). These three sets (OUH-1, −2, −3) were considered different cohorts because of the difference in the experimental reagents of the microarray process.

The characteristics of patients in the training sets and the validation set are shown in Table 1.

**Table 1:** Clinicopathological characteristics of patients in the training sets (OUH-1 and OUH-2) and the validation set (OUH-3)

	Training set (OUH-1 and OUH-2)				Validation set (OUH-3)			
	All patients	ctDNA		*P* value	All patients	ctDNA		*P* value
		Negative	Positive			Negative	Positive	
	(*n* = 35)	(*n* = 25)	(*n* = 10)		(*n* = 13)	(*n* = 12)	(*n* = 1)	
Age (years)								
≤ 50	23	9	3	0.7355	1	1	0	0.7638
> 50	12	16	7		12	11	1	
Tumor diameter								
≤ 20 mm	17	14	3	0.1644	8	7	1	0.4106
> 20 mm	18	11	7		5	5	0	
Histological grade								
1 + 2	29	23	6	0.0658	13	12	1	1.000
3	5	2	3		0	0	0	
ER								
Positive	29	21	8	0.7767	13	12	1	1.000
Negative	6	4	2		0	0	0	
PR								
Positive	24	17	7	0.9612	13	12	1	1.000
Negative	10	7	3		0	0	0	
Unknown	1	0	0					
HER2								
Positive	6	4	2	0.7767	0	0	0	1.000
Negative	29	21	8		13	12	1	
Ki-67								
≥ 20%	16	10	6	0.3828	0	0	0	N.A
< 20%	17	13	4		0	0	0	
Unknown	2	2	0		13	12	1	
Lymphatic invasion								
0	13	9	4	0.9778	0	0	0	N.A
1	16	11	5		0	0	0	
Unknown	6	5	1		13	12	1	
Node metastasis								
Negative	11	8	3	0.9083	13	12	1	1.000
Positive	24	17	7		0	0	0	
Cir-Predict								
Negative	25	25	0	< 0.0001*	13	11	0	0.0146*
Positive	10	0	10		1	2	1	

Statistically significant items are highlited in bold

*ER* estrogen receptor, *PR* progesterone receptor, *HER2* human epidermal growth factor receptor, *N.A.* not available

### RNA extraction and DNA microarray analysis

Tumor tissues were obtained at the time of surgery, immediately snap frozen in liquid nitrogen, and kept at − 80 °C until RNA extraction. The Qiagen RNeasy^®^ mini kit (QIAGEN Science, Germantown, MD, USA) was used to extract RNA from tumor tissues. RNA (200 ng for OUH_1 and 1 µg for OUH_2) was subjected to DNA microarray assay (U133 Plus 2.0 Array; Affymetrix, Santa Clara, CA, USA) following the manufacturer’s instructions. Gene Profiling Reagents (Affymetrix) and One Cycle Target Labeling were used for OUH_1. One Cycle Target Labeling and Control Reagents (Affymetrix) were used for OUH_2 and OUH_3.

### DNA extraction and real-time PCR

DNA was extracted from frozen tumor tissues with the DNeasy Blood & Tissue Kit (QIAGEN, Germantown, MD, USA) following the manufacturer’s instructions. TaqMan-based real-time PCR analysis was conducted to detect the three “hot-spot” PIK3CA mutations (H1047R, E545K, and E542K) using a LightCycler 480 Real-Time PCR System (Roche Applied Science, Mannheim, Germany).

### DNA extraction and digital PCR

DNA was extracted from 500 µl of serum using the QIAamp Circulating Nucleic Acid Kit (QIAGEN, Hilden, Germany) following the manufacturer’s instructions. The DNA was eluted into 50 µl of AVE buffer and stored at − 20 °C. dPCR was performed to detect the three PIK3CA mutations using a QuantStudio™ 3D digital PCR system (Life Technologies, Carlsbad, CA, USA). For the dPCR, 9 µl of template DNA was mixed with 1 µl of 20× TaqMan Assay primer/probe mix and 10 µl of 2× QuantStudio™ 3D Digital PCR Master Mix (Life Technologies) following the manufacturer’s instructions. Fifteen microliter aliquots of the PCR solutions were then loaded into QuantStudio™ 3D Digital PCR 20K chips, and the PCR reaction was performed. The thermal cycler protocol was as follows: 10 min at 96 °C, 39 cycles at 60 °C for 2 min, 98 °C for 30 s, and 60 °C for 1 min. All samples were analyzed in a single assay for each mutation. The data were analyzed with the QuantStudio™ 3D AnalysisSuite™ v1.1.3 (Life Technologies) for mutation search and quantification of the DNA copies in the serum. The mutant allele fraction (MAF, %) was defined as the proportion of mutant DNA copies relative to the sum of mutant and wild-type DNA copies obtained by dPCR. The samples were defined as positive for mutations (ctDNA positive) when one or more mutant alleles were detected per assay and negative (ctDNA negative) when no mutant alleles were detected.

### Immunohistochemical (IHC) assay

ER and PR were expressions defined as positive when 10% or more of the tumor cells were stained by immunohistochemistry (ER: clone 6F11; PR clone 16; Ventana Japan K.K. and SRL Inc. Tokyo, Japan). Human epidermal growth factor receptor 2 (HER2) was examined by immunohistochemistry (anti-human c-erb-2 polyclonal antibody; Nichirei Biosciences, Tokyo, Japan) or by fluorescent in situ hybridization (FISH) using the PathVysion Her2 DNA probe kits (SRL Inc., Tokyo, Japan). For the FISH scoring, the ratio of the HER2 gene signals to the chromosome 17 signals was calculated for each of the specimens. A tumor that exhibited a + 3 immunohistostaining score or a FISH ratio ≥ 2.0 was considered HER2-positive. The histological grade was determined by the Scarff-Bloom-Richardson grading system. Ki-67 was defined as positive when 20% or more of the tumor cells were stained by immunohistochemistry (anti-Ki67 antibody clone 30-9; Roche Applied Science, Mannheim, Germany) as previously described [18]).

### Microarray data processing

The gene expression data sets were obtained by GeneChip™ Human Genome U133 Plus 2.0 Array DNA microarray (Affymetrix). Data were normalized with the Robust Multi-array Average (RMA) procedure [19]. RMA normalization is performed on each dataset (OUH1, 2, 3) individually.

### Statistical analysis

Statistical software R version 3.4.2 (http://www.R-project.org/), Bioconductor packages (http://www.bioconductor.org/), and JMP software were used for statistical analyses. Pathway analysis was analyzed using National Institute of Health DAVID Bioinformatics (https://david.ncifcrf.gov/home.jsp). The associations between the various clinicopathological parameters and cfDNA detection were evaluated with Chi square or Fisher’s exact tests. Univariate and multivariate analyses of the various parameters for the predictions of recurrence and death were conducted with the Cox proportional hazards model.

## Results

### Construction of the Cir-Predict model

We investigated differentially expressed genes between samples with and without ctDNA in the 35 training patient samples (OUH_1 and OUH_2). Of the 54,675 probes, 370 probes have a tendency to be different for the expression levels (*P* < 0.010) depending on whether ctDNA can be detected or not. The metafor Package (A Meta-Analysis Package for R) was used statistically integrate the two training data (OUH_1 and OUH_2) and to rank the differentially expressed genes. Supervised analysis using SVM (support-vector machine) was used for the construction of the prediction model using these probes. The sequential forward filtering method, which assesses the leave-one-out cross-validation, was used to optimize the prediction model. The SVM model comprising 126 probes (111 genes; Supplementary Table S1) exhibited the highest accuracy in the training set (OUH_1 and OUH_2) and was therefore adopted as the prediction model of ctDNA and named Cir-Predict. Cir-Predict was then applied to the validation set (OUH_3) after normalization with the RMA procedure.

Among the 126 probes (111genes), 70 probes were up-regulated and 56 probes were down-regulated in the ctDNA-positive group. The top three ranked genes are *OR10A5* (up-regulated), *EIF3A* (down-regulated), and *STX12* (down-regulated).

The diagnostic accuracy, sensitivity, specificity, positive predictive value (PPV), and negative predictive value (NPV) of Cir-Predict in the training sets (OUH_1 and OUH_2) were100%, 100%, 100%, 100%, and 100%, respectively (Tables 2, 3). The diagnostic accuracy, sensitivity, specificity, PPV, and NPV in the validation set were 92.3%, 100%, 91.7%, 50%, and 100%, respectively (Tables 2, 3).

**Table 2:** The contingency table of Cir-Predict

		Training set (*n* = 35)		Validation set (*n* = 13)	
		Cir-Predict		Cir-Predict	
		(−)	(+)	(−)	(+)
Actual ctDNA Detection	(−)	25	0	11	0
	(+)	0	10	1	1

**Table 3:** The diagnostic accuracy, sensitivity, specificity, PPV, and NPV of Cir-Predict

	Training set (*n* = 35)	Validation set (*n* = 13)
Accuracy (%)	100	92.3
Sensitivity (%)	100	100
Specificity (%)	100	91.7
PPV (%)	100	50
NPV (%)	100	100

### Univariate and multivariate analyses of Cir-Predict in the prediction of ctDNA detection

We next examined the association of various clinicopathological parameters and Cir-Predict with ctDNA detection in the training sets and validation set (Table 4).

**Table 4:** Univariate and multivariate analyses of parameters associated with ctDNA detectability in the training sets and the validation set

	Training set				Validation set			
	Univariate analysis		Multivariate analysis		Univariate analysis		Multivariate analysis	
	Odds ratio (95%CI)	*P* value	Odds ratio (95%CI)	*P* value	Odds ratio (95%CI)	*P* value	Odds ratio (95%CI)	*P* value
Age (years)	1.31	0.74	1	1.00	N.A	0.76	1.24E–14	1.00
> 50 vs. ≤ 50	(0.27–6.37)							
Tumor diameter (mm)	2.97	0.16	1	1.00	N.A	0.41	7.04E–15	0.10
> 20 vs. ≤ 20	(0.62–14.22)							
Histological grade	5.75	0.07	1	1.00	N.A	1.00	N.A	N.A
3 vs. 1 + 2	(0.78–42.58)							
ER	0.76	0.78	1	1.00	N.A	1.00	N.A	N.A
(+) vs. (−)	(0.12–5.01)							
PR	0.96	0.96	1	1.00	N.A	1.00	N.A	N.A
(+) vs. (−)	(0.19–4.82)							
HER2	1.31	0.78	1	1.00	N.A	N.A	N.A	N.A
(+) vs. (−)	(0.20–8.62)							
Ki-67	1.95	0.38	1	1.00	N.A	N.A	N.A	N.A
(+) vs. (–)	(0.43–8.83)							
Lymphatic invasion	1.02	0.98	1	1.00	N.A	N.A	N.A	N.A
Ly1 vs. Ly0	(0.21–4.98)							
Node metastasis	1.10	0.91	1	1.00	N.A	1.00	N.A	N.A
(+) vs. (−)	(0.22–5.40)							
Cir-Predict	N.A	< 0.0001*	6.47E+15	< 0.0001*	N.A	0.0146*	5.46E + 14	0.0141*
(+) vs. (−)								

Statistically significant items are highlited in bold

*CI* confidence interval, *ER* estrogen receptor, *PR* progesterone receptor, *HER2* human epidermal growth factor receptor, *N.A.* not available

Univariate analysis showed that Cir-Predict was most significantly associated with ctDNA detection in the training set (*P* < 0.001) and validation set (*P* = 0.0146). Multivariate analysis showed that Cir-Predict was most significantly associated with ctDNA detection in the training set and validation set independently of the other parameters (*P* < 0.001, *P* = 0.0141, respectively).

### Visualization of Cir-Predict gene expression by gene clustering analysis (heat map)

**Fig. 1:**
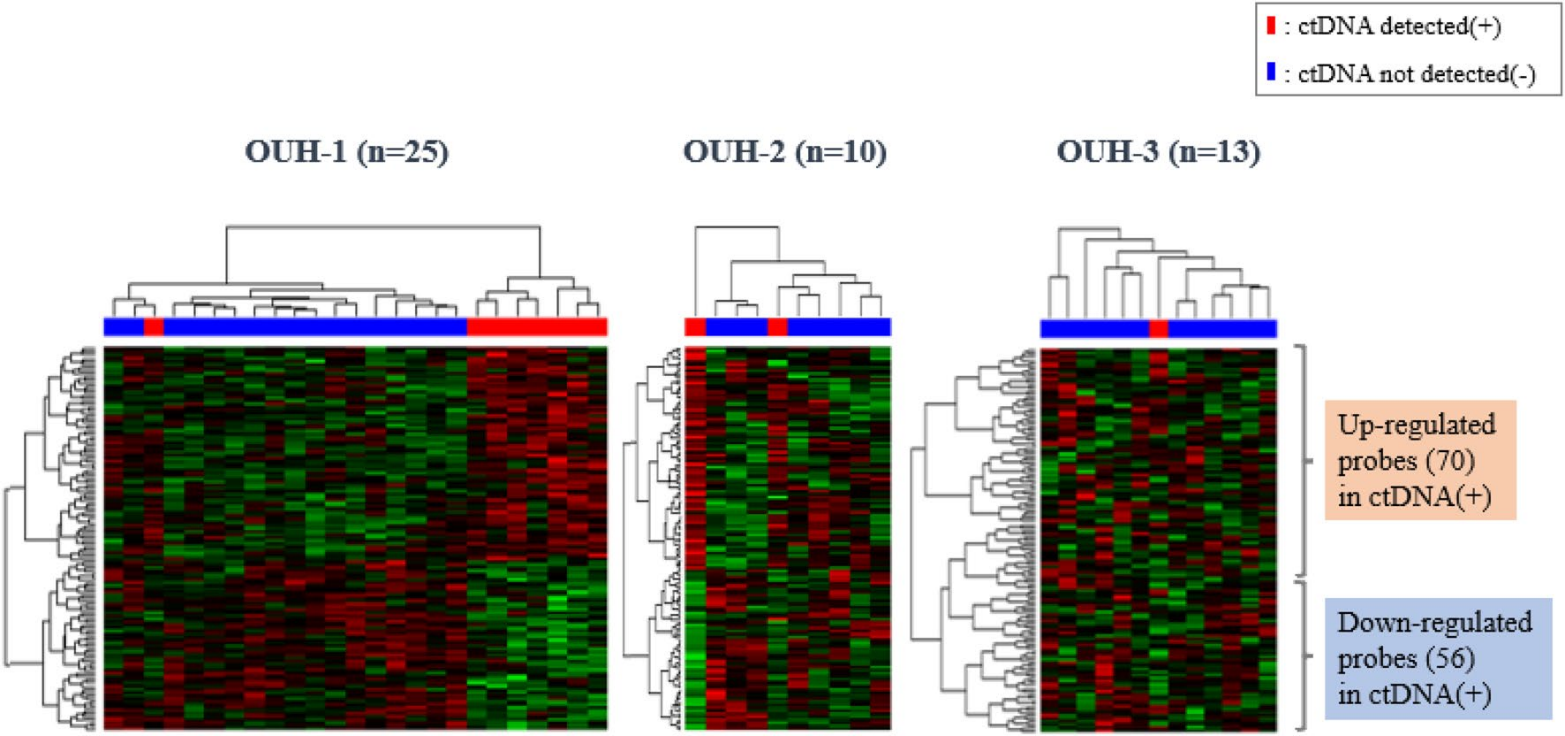
Differentially expressed genes in breast cancer samples with and without detected ctDNA. The heat map for the training set (OUH-1, *n* = 25), the heat map for the training set (OUH-2, *n* = 10), and the heat map for the validation set (OUH-3, *n* = 13)

Clustered heat maps were built for visualization and interpretation of genome-scale molecular profiling data. Among the 126 probes in the Cir-Predict, 70 probes had increased expression, and 56 probes had decreased expression in the ctDNA-positive group. The gene expression tendency was the same in the validation set and the training sets (Fig. 1).

### Pathway analysis

Kyoto Encyclopedia of Genes and Genomes (http://www.genome.jp//kegg/pathway.html)^20^ pathway enrichment analysis was performed of the 370 probes used as the source of genes for the construction of Cir-Predict. Nine pathways were significantly associated with ctDNA (*P* < 0.05; Table 5). The tight junction pathway was the most strongly associated (*P* = 3.85E−3) followed by the ubiquitin mediated proteolysis pathway (*P* = 5.27E−03). Previous studies indicated that the cell cycle and junction function are related to ctDNA. In our analysis, the cell cycle pathway was the seventh most strongly associated with ctDNA (*P* = 2.99E−2) and the adherens junction pathway was the ninth most strongly associated (*P* = 4.97E−2). Among the 370 probes, 9 genes (*JUN*, *NEDD4L*, *RAP1A*, *RAC1*, *ARHGEF2*, *ACTR3*, *HSPA4*, *PARD3*, *STK11*) were involved in the tight junction pathway.

**Table 5:** KEGG pathway analysis of pathways significantly associated with ctDNA detectability

	Pathway	Count	%	*P* value	Pop Hits	Fold Enr	Bonf	Benj	FDR
1	hsa04530: Tight junction	9	3.02	3.85E−03	170	3.52	0.57	0.4	0.4
2	hsa04120: Ubiquitin mediated proteolysis	8	2.68	5.27E−03	142	3.75	0.69	0.4	0.4
3	hsa03013: Nucleocytoplasmic transport	7	2.35	5.44E−03	108	4.31	0.7	0.4	0.4
4	hsa05210: Colorectal cancer	6	2.01	9.10E−03	86	4.64	0.87	0.47	0.47
5	hsa05161: Hepatitis B	8	2.68	1.06E−02	162	3.28	0.9	0.47	0.47
6	hsa04972: Pancreatic secretion	6	2.01	1.80E−02	102	3.91	0.98	0.66	0.66
7	hsa04110: Cell cycle	7	2.35	2.99E−02	157	2.96	1.00	0.94	0.94
8	hsa03040: Spliceosome	8	2.68	4.26E−02	216	2.46	1.00	1.00	1.00
9	hsa04520: Adherens junction	5	1.68	4.97E−02	93	3.58	1.00	1.00	1.00

*Fold Enr.* fold enrichment, *Bonf.* Bonerroni, *Benj.* Benjamini, *FDR* false discovery rate

### Overlapping genes with conventional prognosis prediction models

Gene expression assays provide prognostic and therapy-predictive information that complements biomarker information. The 21-gene assay (Oncotype Dx) is preferred by the NCCN Breast Cancer Panel for the prognosis and prediction of chemotherapy benefit [21, 22]. Previous studies reported that the presence of ctDNA is related to poor prognosis in primary breast cancer patients [4, 5]. We examined whether the genes in Cir-Predict overlapped with the 21 genes in the assay, and we identified only one overlapping gene, Bcl2 (207005_s_at).

### Prognostic value of Cir-Predict for recurrence-free survival in breast cancer patients

**Fig. 2:**
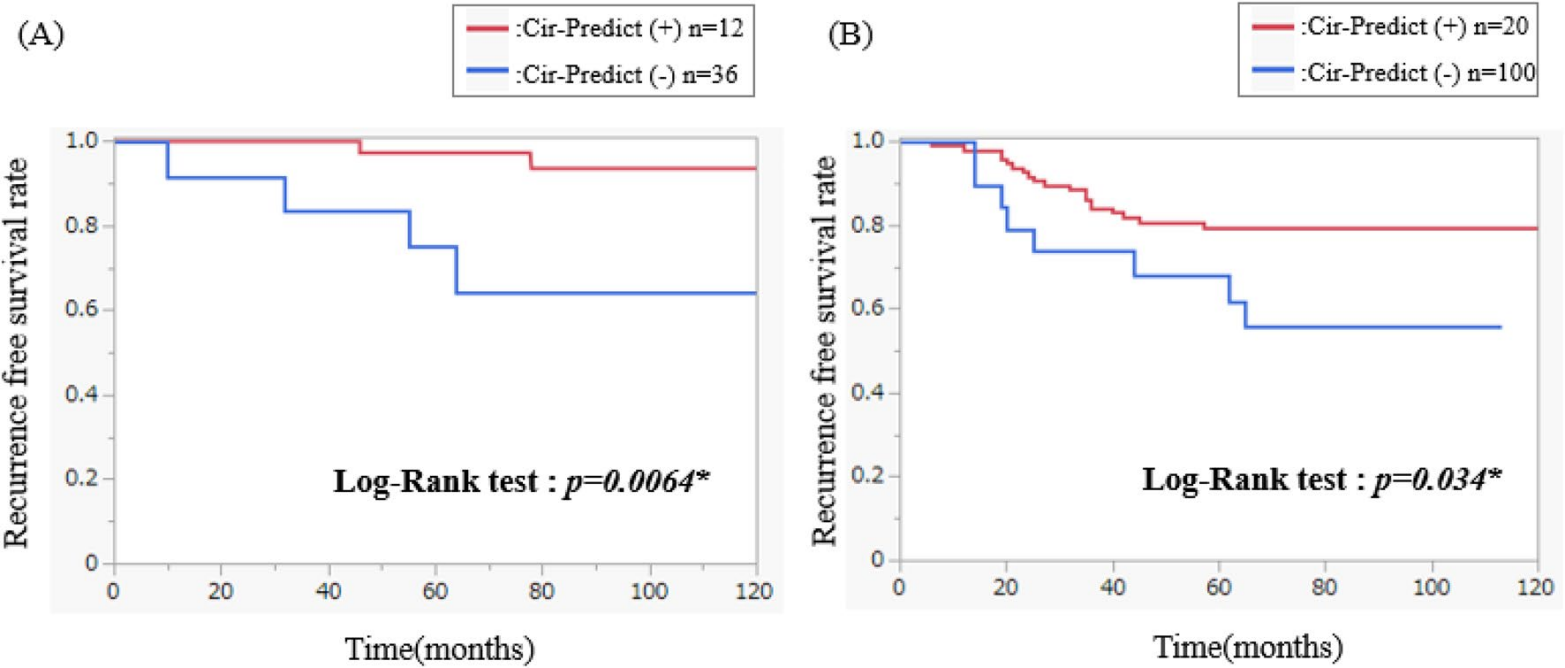
Kaplan–Meier (KM) estimates of recurrence-free survival (RFS) based on Cir-Predict. **A** In a combined cohort of the training and the validation set (*n* = 38). **B** In a cohort of ER+/HER2−/n0 invasive breast cancer patients (*n* = 120)

First, to evaluate the prognostic significance of Cir-Predict, survival analysis was performed in a combined cohort of the training and the validation set (*n* = 38). They were followed up for a median of 102.0 (range, 10–147 months) postoperative months. Of the 38 patients, 12 patients were classified as Cir-Predict (+) and 26 patients were classified as Cir-Predict (−). Kaplan–Meier survival analysis demonstrated a statistically significant difference between the two groups, Cir-Predict (+) and Cir-Predict (−), (*P* = 0.0064, log-rank test) in relapse-free survival (RFS) of 38 patients (Fig. 2A).

Secondly, given the established association between ctDNA prevalence and breast cancer subtype, and the known prognostic variability across subtypes, to mitigate the potential confounding effect of subtype-associated prognosis, survival analysis was performed in a cohort of estrogen receptor (ER)-positive, human epidermal growth factor receptor 2 (HER2)-negative and node-negative (ER+/HER2−/n0) invasive breast cancer patients (*n* = 120). This cohort was used in our institution to validate a prognostic prediction model for ER-positive breast cancer in the previous study [15, 23]. The mean age was 55.5 and all of patients had invasive ductal carcinoma with clinical T1 (*n* = 36, 30%), T2 (*n* = 80, 66.6%), T3 (*n* = 4, 3.3%) node-negative disease. They were followed up for a median of 73.5 (range, 5–126) postoperative months. Of the 120 patients, 20 patients were classified as Cir-Predict (+) and 100 patients were classified as Cir-Predict (–). Log-rank test was used for comparisons between groups and Kaplan–Meier analysis used for survival outcomes. With the Cir-Predict we could classify the ER+/HER2−/n0 120 patients into a positive (*n* = 20) and a negative (*n* = 100) group with recurrence-free survival rates (RFS) of 81% and 60%, respectively (*P* = 0.03) (Fig. 2B).

## Discussion

In this present study, we constructed the Cir-Predict model, which can classify breast cancers into the ctDNA-positive group and ctDNA-negative group, and demonstrated its success in the training sets and the validation set. The amount of ctDNA is affected by many factors. Our results showed that Cir-Predict, comprising differentially expressed genes, was related to ctDNA detectability; this indicates that tumor gene expression plays a major role in ctDNA detectability.

The Cir-Predict can predict the detection of ctDNA, which helps us to know the cause whether ctDNA not included in the cfDNA or not. The information helps in the understanding of the results of cfDNA-based CGP assays, especially when there is a discrepancy between the result of cfDNA-based CGP assays and the result of tumor tissue-based CGP assays.

Pathway analysis of the probe set source for the construction of the Cir-Predict indicated that breakdown of junctions and cell cycle were associated with differentially expressed genes in ctDNA-positive samples. Tight junctions are intercellular adhesion complexes in epithelia and endothelia that control paracellular permeability, which support the maintenance of cell polarity by restricting intermixing of apical and basolateral transmembrane components [24]. Cell–cell adherens junctions are the most common type of intercellular adhesions and are important for maintaining tissue architecture and cell polarity [25]. In the adhesion between epithelial cells, three structures, tight junction, adherens junction, and desmosome, play the main role. These structures enable selective permeability of substances between cells and develop epithelial cell polarity between the cell membrane in contact with the extracellular space and the cell membrane in contact with the internal environment [26, 27]. The breakdown of tight junction and adherens junction pathways may mean breakdown of epithelial cell structure, function, and polarity.

The top three ranked genes in the Cir-Predict are *OR10A5* (up-regulated), *EIF3A* (down-regulated), and *STX12* (down-regulated). Recent reports indicated that *EIF3A* functions in various cancers. * EIF3a* is the largest subunit of *EIF3*, which is a key player in all steps of translation initiation. *EIF3a* is suggested to be correlated with cancer occurrence, metastasis, prognosis, and therapeutic response [28].

There was only one overlapping gene between the Cir-Predict and Oncotype Dx: the *Bcl2* gene (down-regulated). *Bcl2* is involved in the regulation of apoptosis. Antisense drugs (antisense oligonucleotides) that inactivate *Bcl-2* mRNA to prevent the production protein have been developed for breast cancer treatments [29].

There are many genes whose functions have not yet been clarified among the genes in Cir-Predict. Our results indicate that Cir-Predict may be able to predict the detectability of ctDNA independently of other factors. However, each gene needs to be studied to understand the mechanism of ctDNA secretion into blood. Elucidating the functions of these genes may help clarify the mechanism by which ctDNA appears in the blood.

Cir-Predict demonstrated its ability to predict recurrence not only in the data used to develop the model but also in ER+/HER2− breast cancer patients, supporting the prior reports that ctDNA presence is a poor prognostic factor. The finding that Cir-Predict is predictive of recurrence in ER+/HER2− breast cancer implies that the presence of ctDNA, regardless of its low prevalence, may be an indicator of poor prognosis in this ER+/HER2− subtype.

## Supplementary Information

Below is the link to the electronic supplementary material.

Supplementary file1 (DOCX 42 KB)

## Data Availability

The datasets generated and analyzed during the current study are available from the corresponding author on reasonable request.

## Abbreviations

ctDNA: Circulating tumor DNA
dPCR: Digital PCR
CGP: CfDNA-based comprehensive genomic profiling
