# Supplementary material for: Development of a prediction model for ctDNA detection (Cir-Predict) in breast cancer
Source: Breast Cancer Res Treat. 2025 Mar 7;211(2):331–9. doi: 10.1007/s10549-025-07647-0 (PMC12006266; doi:10.1007/s10549-025-07647-0)
Supplement: Supplementary file 1 — Supplementary file1 (DOCX 42 KB) [file 10549_2025_7647_MOESM1_ESM.docx]

**Supplementary Table 1.** List of the genes of Cir-Predict.

| Ranking | Probe Set ID | p_val | effect_size | Entrez Gene | UniGene ID | Gene Symbol | Up/downregulation in the ctDNA(+) group |
| --- | --- | --- | --- | --- | --- | --- | --- |
| 1 | 1555651_at | 1.63E-05 | 0.442543215 | 144124 | Hs.447478 | *OR10A5* | up |
| 2 | 200597_at | 2.43E-05 | 0.43961196 | 8661 | Hs.523299 | *EIF3A* | down |
| 3 | 212111_at | 4.87E-05 | 0.431755563 | 23673 | Hs.523855 | *STX12* | down |
| 4 | 205027_s_at | 1.43E-04 | 0.454748908 | 1326 | Hs.432453 | *MAP3K8* | down |
| 5 | 212072_s_at | 1.93E-04 | 0.424994475 | 1457 /// 283106 | Hs.644056 | *CSNK2A1 /// CSNK2A1P* | up |
| 6 | 244148_at | 1.97E-04 | 0.421153451 | --- | --- | *---* | up |
| 7 | 228223_at | 2.27E-04 | 0.424554554 | 140831 | Hs.292135 | *ZSWIM3* | up |
| 8 | 208617_s_at | 2.39E-04 | 0.419891422 | 8073 | Hs.470477 | *PTP4A2* | down |
| 9 | 1555682_at | 2.43E-04 | 0.42096889 | 78990 | Hs.278815 | *OTUB2* | up |
| 10 | 211042_x_at | 2.86E-04 | 0.419403971 | 4162 | Hs.599039 | *MCAM* | up |
| 11 | 203625_x_at | 2.97E-04 | 0.419752492 | 6502 | Hs.23348 | *SKP2* | up |
| 12 | 208615_s_at | 3.18E-04 | 0.419171111 | 8073 | Hs.470477 | *PTP4A2* | down |
| 13 | 1558237_x_at | 3.24E-04 | 0.417840756 | --- | --- | *---* | down |
| 14 | 1556976_s_at | 3.60E-04 | 0.4173172 | --- | Hs.616647 | *---* | up |
| 15 | 244649_at | 3.96E-04 | 0.417370649 | 646484 | Hs.733937 | *LOC646484* | up |
| 16 | 224745_x_at | 4.51E-04 | 0.416305388 | 55593 | Hs.496098 | *OTUD5* | up |
| 17 | 225966_at | 4.57E-04 | 0.415181754 | 284184 | Hs.356545 | *C17orf89* | down |
| 18 | 209926_at | 4.85E-04 | 0.415090352 | 729991 | Hs.153629 | *MEF2BNB* | up |
| 19 | 1558236_at | 4.91E-04 | 0.414937833 | --- | --- | *---* | down |
| 20 | 228400_at | 5.30E-04 | 0.415531007 | 57619 | Hs.702168 | *SHROOM3* | down |
| 21 | 213508_at | 5.35E-04 | 0.414908072 | 171546 | Hs.740577 | *SPTSSA* | down |
| 22 | 202815_s_at | 5.81E-04 | 0.413813759 | 10614 | Hs.702240 | *HEXIM1* | down |
| 23 | 217738_at | 6.86E-04 | 0.415236847 | 10135 | Hs.489615 | *NAMPT* | down |
| 24 | 213574_s_at | 7.47E-04 | 0.413570536 | --- | Hs.595245 | *---* | down |
| 25 | 231296_at | 7.55E-04 | 0.412408078 | --- | --- | *---* | down |
| 26 | 209068_at | 7.83E-04 | 0.412573742 | 9987 | Hs.656910 | *HNRPDL* | down |
| 27 | 232340_at | 7.84E-04 | 0.412196333 | 100507637 | Hs.642767 | *LOC100507637* | down |
| 28 | 221207_s_at | 8.08E-04 | 0.411405405 | 26960 | Hs.491172 | *NBEA* | down |
| 29 | 201018_at | 8.42E-04 | 0.411621141 | 1964 | Hs.522590 | *EIF1AX* | down |
| 30 | 216290_x_at | 8.57E-04 | 0.415202258 | --- | Hs.675436 | *---* | down |
| 31 | 214211_at | 8.99E-04 | 0.410749526 | 2495 | Hs.524910 | *FTH1* | down |
| 32 | 212707_s_at | 9.18E-04 | 0.411181754 | 100271927 /// 10156 /// 401331 | Hs.632627 | *RASA4 /// RASA4B /// RASA4CP* | up |
| 33 | 207005_s_at | 9.42E-04 | 0.418939075 | 596 | Hs.150749 | *BCL2* | down |
| 34 | 220348_at | 9.61E-04 | 0.414773415 | 114818 | Hs.130593 | *KLHL29* | up |
| 35 | 225145_at | 1.05E-03 | 0.409696645 | 57727 | Hs.654991 | *NCOA5* | up |
| 36 | 232419_at | 1.07E-03 | 0.409580174 | 54972 | Hs.118552 | *TMEM132A* | up |
| 37 | 236716_at | 1.10E-03 | 0.415226923 | --- | Hs.254117 | *---* | up |
| 38 | 201536_at | 1.19E-03 | 0.414538816 | 1845 | Hs.181046 | *DUSP3* | down |
| 39 | 217834_s_at | 1.19E-03 | 0.413047619 | 10492 | Hs.571177 | *SYNCRIP* | up |
| 40 | 227717_at | 1.24E-03 | 0.408648378 | 389337 | Hs.256206 | *ARHGEF37* | down |
| 41 | 1559121_s_at | 1.28E-03 | 0.41063199 | --- | Hs.662634 | *---* | down |
| 42 | 208598_s_at | 1.29E-03 | 0.410137258 | 10075 | Hs.136905 | *HUWE1* | up |
| 43 | 205624_at | 1.40E-03 | 0.407874969 | 1359 | Hs.646 | *CPA3* | down |
| 44 | 224711_at | 1.41E-03 | 0.409386677 | 7528 | Hs.388927 | *YY1* | down |
| 45 | 225130_at | 1.42E-03 | 0.540877869 | 54764 | Hs.595158 | *ZRANB1* | down |
| 46 | 1559909_a_at | 1.43E-03 | 0.408380962 | 10245 | Hs.30570 | *TIMM17B* | up |
| 47 | 225641_at | 1.46E-03 | 0.411248488 | 4209 | Hs.314327 | *MEF2D* | down |
| 48 | 220635_at | 1.51E-03 | 0.407570296 | 170680 | Hs.146824 | *PSORS1C2* | up |
| 49 | 234847_at | 1.52E-03 | 0.408942245 | 150992 | --- | *LINC00309* | up |
| 50 | 217345_at | 1.53E-03 | 0.407323216 | --- | Hs.638422 | *---* | up |
| 51 | 241768_at | 1.57E-03 | 0.407392754 | --- | --- | *---* | down |
| 52 | 242766_at | 1.57E-03 | 0.40769537 | --- | Hs.558872 | *---* | up |
| 53 | 1569931_at | 1.61E-03 | 0.408060999 | --- | Hs.676055 | *---* | up |
| 54 | 226209_at | 1.64E-03 | 0.40866732 | 4731 | Hs.473937 | *NDUFV3* | up |
| 55 | 211342_x_at | 1.68E-03 | 0.407746257 | 9968 | Hs.409226 | *MED12* | up |
| 56 | 225468_at | 1.72E-03 | 0.406809184 | 219988 | Hs.591960 | *PATL1* | up |
| 57 | 225553_at | 1.73E-03 | 0.407807554 | --- | Hs.594424 | *---* | down |
| 58 | 226439_s_at | 1.77E-03 | 0.406502424 | 26960 | Hs.491172 | *NBEA* | down |
| 59 | 224609_at | 1.80E-03 | 0.406320375 | 57153 | Hs.534560 | *SLC44A2* | up |
| 60 | 208938_at | 1.81E-03 | 0.40639877 | 5546 | Hs.516948 | *PRCC* | up |
| 61 | 210315_at | 1.83E-03 | 0.406907311 | 6854 | Hs.445503 | *SYN2* | up |
| 62 | 216071_x_at | 1.87E-03 | 0.406816294 | 9968 | Hs.409226 | *MED12* | up |
| 63 | 206684_s_at | 2.01E-03 | 0.405599692 | 11016 | Hs.12286 | *ATF7* | up |
| 64 | 223978_s_at | 2.05E-03 | 0.405477954 | 54675 | Hs.224764 | *CRLS1* | up |
| 65 | 218619_s_at | 2.11E-03 | 0.405371655 | 6839 | Hs.522639 | *SUV39H1* | up |
| 66 | 239104_at | 2.14E-03 | 0.406123029 | 439933 | Hs.591071 | *MGC42157* | down |
| 67 | 229427_at | 2.17E-03 | 0.407654579 | 9037 | Hs.27621 | *SEMA5A* | down |
| 68 | 220513_at | 2.18E-03 | 0.405822801 | 100129128 | Hs.663639 | *KHDC1L* | up |
| 69 | 1553873_at | 2.18E-03 | 0.405184322 | 257240 | Hs.448572 | *KLHL34* | up |
| 70 | 57703_at | 2.21E-03 | 0.407410012 | 205564 | Hs.240770 | *SENP5* | up |
| 71 | 216499_at | 2.22E-03 | 0.40580551 | --- | Hs.409816 | *---* | up |
| 72 | 203578_s_at | 2.22E-03 | 0.405076625 | 9057 | Hs.679580 | *SLC7A6* | up |
| 73 | 229958_at | 2.24E-03 | 0.405134039 | 2055 | Hs.127675 | *CLN8* | down |
| 74 | 212378_at | 0.002241 | 0.40585035 | 2618 | Hs.473648 | GART | up |
| 75 | 215657_at | 0.00227 | 0.408486065 | 1811 | Hs.1650 | SLC26A3 | up |
| 76 | 218131_s_at | 0.002309 | 0.405377227 | 54815 | Hs.118964 | GATAD2A | up |
| 77 | 1560806_at | 0.002321 | 0.409472269 | 150527 /// 646743 | Hs.125706 | LOC646743 /// TISP43 | up |
| 78 | 228281_at | 0.002331 | 0.409294552 | 220042 | Hs.165607 | C11orf82 | up |
| 79 | 236655_at | 0.002339 | 0.404734793 | 7163 | Hs.368433 | TPD52 | down |
| 80 | 230010_at | 0.002386 | 0.405475392 | 84530 | Hs.112577 | SRRM4 | up |
| 81 | 236441_at | 0.002411 | 0.405703171 | --- | Hs.127116 | --- | up |
| 82 | 230051_at | 0.002425 | 0.408857047 | 254427 | Hs.435775 | C10orf47 | down |
| 83 | 216960_s_at | 0.002433 | 0.405534078 | 7692 | Hs.472221 | ZNF133 | up |
| 84 | 213803_at | 0.002443 | 0.405202212 | --- | Hs.595245 | --- | down |
| 85 | 242919_at | 0.002451 | 0.406620548 | 56242 | Hs.301059 | ZNF253 | up |
| 86 | 227034_at | 0.002457 | 0.404530911 | 65124 | Hs.355455 | SOWAHC | down |
| 87 | 222478_at | 0.002485 | 0.404321227 | 51028 | Hs.109520 | VPS36 | down |
| 88 | 232910_at | 0.002492 | 0.409136614 | 92482 | --- | BBIP1 | down |
| 89 | 235555_at | 0.002527 | 0.414825433 | --- | Hs.48729 | --- | up |
| 90 | 1563709_at | 0.002546 | 0.404212672 | 100127955 /// 100128374 | Hs.683855 | LOC100127955 /// LOC100128374 | up |
| 91 | 203486_s_at | 0.002552 | 0.405414709 | 25852 | Hs.740399 | ARMC8 | up |
| 92 | 218894_s_at | 0.002582 | 0.407478147 | 55110 | Hs.104650 | MAGOHB | up |
| 93 | 229276_at | 0.002587 | 0.404577104 | 57549 | Hs.591472 | IGSF9 | up |
| 94 | 230005_at | 0.00259 | 0.404331357 | 258010 | Hs.349096 | SVIP | down |
| 95 | 224210_s_at | 0.002599 | 0.404737687 | 11264 | Hs.654857 | PXMP4 | down |
| 96 | 236074_at | 0.002766 | 0.405441959 | 100506676 | --- | LOC100506676 | up |
| 97 | 216315_x_at | 0.002777 | 0.403627247 | --- | --- | --- | up |
| 98 | 208920_at | 0.002811 | 0.406447537 | 6717 | Hs.489040 | SRI | down |
| 99 | 238746_at | 0.002813 | 0.403757047 | 11264 | Hs.654857 | PXMP4 | down |
| 100 | 1565585_at | 0.002832 | 0.406269328 | --- | Hs.641182 | --- | up |
| 101 | 234444_at | 0.002845 | 0.403591515 | --- | Hs.610980 | --- | up |
| 102 | 206597_at | 0.002882 | 0.403784648 | 4901 | Hs.652297 | NRL | up |
| 103 | 1561386_at | 0.002927 | 0.40335666 | --- | Hs.588388 | --- | up |
| 104 | 226163_at | 0.00293 | 0.403307142 | 221504 | Hs.591805 | ZBTB9 | up |
| 105 | 224484_s_at | 0.003061 | 0.403744636 | 84312 | Hs.525299 | BRMS1L | up |
| 106 | 208640_at | 0.003083 | 0.403094811 | 5879 | Hs.413812 | RAC1 | down |
| 107 | 212553_at | 0.003094 | 0.403824396 | 23248 | Hs.213666 | RPRD2 | up |
| 108 | 207558_s_at | 0.00311 | 0.403639899 | 5308 | Hs.643588 | PITX2 | up |
| 109 | 242384_at | 0.003184 | 0.403368781 | --- | Hs.605187 | --- | down |
| 110 | 201746_at | 0.003198 | 0.403443668 | 7157 | Hs.437460 | TP53 | up |
| 111 | 212058_at | 0.003202 | 0.407487772 | 23350 | Hs.596572 | U2SURP | up |
| 112 | 230551_at | 0.003242 | 0.402814908 | 283455 | Hs.375836 | KSR2 | down |
| 113 | 219193_at | 0.003246 | 0.402763036 | 55100 | Hs.213690 | WDR70 | up |
| 114 | 225000_at | 0.003257 | 0.402710208 | 5576 | Hs.631923 | PRKAR2A | down |
| 115 | 215812_s_at | 0.00326 | 0.403447957 | 386757 /// 6535 /// 653562 | Hs.540696 | LOC653562 /// SLC6A10P /// SLC6A8 | up |
| 116 | 236016_at | 0.003272 | 0.404826037 | 6605 | Hs.740388 | SMARCE1 | down |
| 117 | 215491_at | 0.003291 | 0.403333915 | 4610 | Hs.437922 | MYCL1 | up |
| 118 | 225198_at | 0.003306 | 0.405298043 | 9218 | Hs.165195 | VAPA | down |
| 119 | 1558834_s_at | 0.003314 | 0.402653536 | 254268 | Hs.740704 | AKNAD1 | down |
| 120 | 224829_at | 0.003329 | 0.402943954 | 80315 | Hs.127126 | CPEB4 | down |
| 121 | 213573_at | 0.003361 | 0.402712598 | --- | Hs.595245 | --- | down |
| 122 | 1561624_at | 0.00338 | 0.405539096 | --- | Hs.385774 | --- | up |
| 123 | 1564580_at | 0.003393 | 0.402507535 | --- | Hs.671960 | --- | down |
| 124 | 224755_at | 0.003398 | 0.403106149 | 56889 | Hs.500674 | TM9SF3 | down |
| 125 | 212369_at | 0.003399 | 0.40262282 | 171017 | Hs.103315 | ZNF384 | up |
| 126 | 226226_at | 0.00341 | 0.402640482 | 120224 | Hs.504301 | TMEM45B | down |
